# The role of cardiac imaging in assessing the cardiac involvement of type 1 Gaucher disease: a case report with review of literature

**DOI:** 10.1186/s43044-024-00465-7

**Published:** 2024-03-24

**Authors:** Ahmed Youssouf Addou, Wafa El Mire, Nawal Doghmi, Aatif Benyass

**Affiliations:** 1 Department of Cardiology, Cheikh Zaid International University Hospital, Rabat, Morocco; 2 Abulcasis International University of Health Sciences, Rabat, Morocco; 3 Cardiology B Department of CHU Ibn Sina, Mohammed V University, Rabat, Morocco

**Keywords:** Gaucher disease, Syncope, Echocardiography, CMR, Myocardial interstitial fibrosis

## Abstract

**Background:**

Gaucher disease (GD) is a lysosomal storage disease that leads to the accumulation of glucocerebroside within reticuloendothelial cells, haematological, neurological, skeletal and abdominal organs. These clinical manifestations are common to all types of GD, but categorization depends on the absence of neurological involvement (type I) or its presence (type II and III). Cardiac involvement is rare and only reported in few cases, where valvular and aortic calcifications were associated with type IIIc. Other cardiac manifestations, such as constrictive pericarditis, pulmonary hypertension, myocardial infiltration, and restrictive cardiomyopathy, had also been reported.

**Case presentation:**

We report a case of a 72-year-old patient with known type 1 GD who presented with a sudden syncope during exercise. He reported also an exercise intolerance evolving for three months. Echocardiography found concentric left ventricular hypertrophy with segmental hypokinesis, bi-atrial enlargement, and mildly reduced ejection fraction. Mitral flow was in favour of grade II diastolic dysfunction with elevated filling pressure. Cardiac magnetic resonance (CMR) showed interstitial fibrosis in the basal infero-septal wall, probably due to the myocardial infiltration of GD. Due to the lack of echocardiographic and CMR hallmarks of cardiac GD, we conducted a literature review on similar findings.

**Conclusion:**

This case illustrates the importance of non-invasive cardiac imaging in the diagnosis, prognosis and management of cardiac manifestations of GD.

## Background

Gaucher disease is a lysosomal storage disorder that results with the accumulation of glucosylceramide in the cells of reticuloendothelial tissue. This global accumulation leads to hepatosplenomegaly, thrombocytopenia, anaemia, neurologic signs and many inconsistent clinical manifestations. Cardiac involvement is rare and typically seen in type IIIc GD associated with extensive valvular and aortic calcifications [1]. In this article, we report a case of 72-year-old patient with known type 1 GD who presented a syncope. Our study aims to evaluate the cardiovascular involvement of GD, such as myocardial infiltration and ventricular function abnormalities, using echocardiography and cardiac magnetic resonance (CMR).

## Case presentation

A 72-year-old male patient, a retired factory worker, who had no cardiovascular risk factor beside sex and age, had a history of undocumented dysthyroidia. He was diagnosed with type 1 Gaucher disease 1 year prior to current hospital admission. A bone marrow biopsy found Gaucher cells, and a reduced glucocerebrosidase activity was detected. The patient suffered from a syncope that occurred during mild physical effort 4 days before admission. He described also a progressive onset of exercise intolerance and bilateral lower limb oedema over the last three months. He denied any other cardiac symptoms. On admission, his blood pressure was 126/86 mmHg, with a heart rate of 124 bpm. Cardiac auscultation found irregular rhythm, without murmurs or gallops. A Jugular venous distension, a hepatojugular reflux and a bilateral lower limb oedema limited to ankles were observed. Abdominal examination revealed hepatosplenomegaly. A mobile soft thyroid nodule was found with no signs of thyrotoxicosis or cervical adenopathy.

**Fig. 1:**
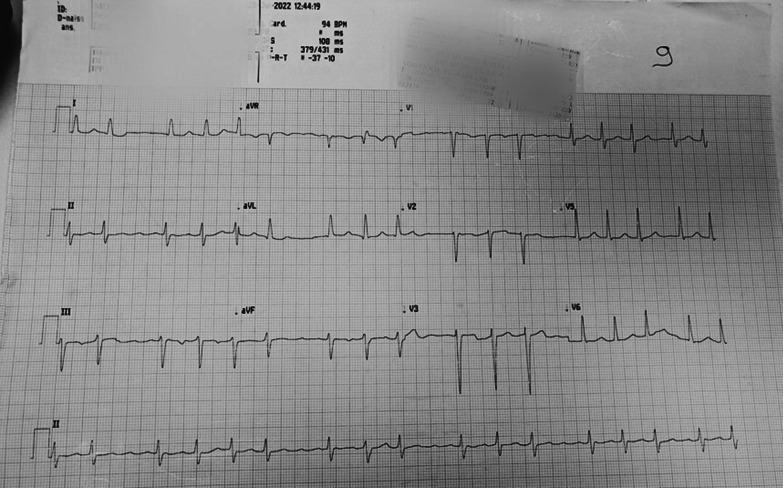
Electrocardiogram showing focal atrial tachycardia at 94bpm, left axis, left ventricular hypertrophy, a poor R antero-septal progression and repolarization abnormalities in inferior leads

The electrocardiogram showed focal atrial tachycardia, left axis, left ventricular hypertrophy, a poor R antero-septal progression and repolarization abnormalities in inferior leads **(**Fig. 1**)**. The pattern of atrioventricular conduction with “ the grouped beating” was in favour of focal atrial tachycardia. The laboratory workup showed a bi-cytopenia (haemoglobin level at 13 g/dl, white cell counts at 3130/mm^3^, platelets at 54,000/mm^3^), mild hepatic cytolysis, BNP level at 590 pg/ml and a very low TSH at 0,07 UI/L with a high free T4 level at 1.94 ng/dL. Chest X-ray showed signs of pulmonary interstitial oedema of cardiac origin.

**Fig. 2:**
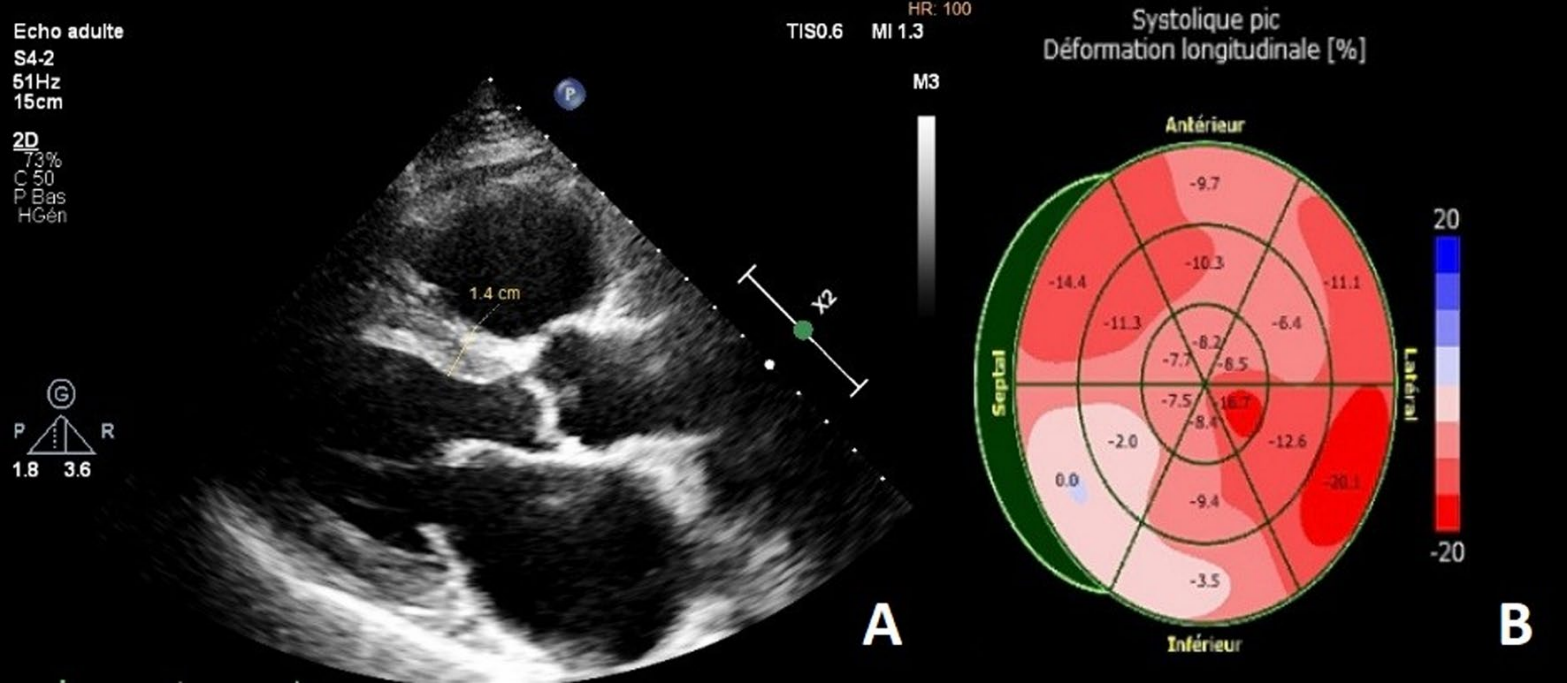
Transthoracic echocardiography revealing concentric hypertrophy with mildly reduced LVEF (43%). **A** The parasternal long-axis view shows an enlarged left atrium, normal valves structure with no calcification, septal and posterior wall hypertrophy. **B** The bull’s-eye plot from two-dimensional speckle-tracking echography visualizing the regional wall motion abnormalities and reduced GLS 9.6%

Echocardiography demonstrated a concentric left ventricular hypertrophy (LVH) with global hypokinesis and marked wall motion disturbances in the basal and median segments of the infero-septal and inferior walls. The systolic function was mildly impaired ejection fraction at 43%. The global longitudinal strain was reduced (− 9.6%) with regional wall motion abnormalities **(**Fig. 2b**)**. Mitral and aortic valves had normal structure with minimal mitral regurgitation **(**Fig. 2a**).** No ejection tract obstruction was found. Bi-atrial enlargement (Left atrium volume at 52 ml/m^2^; Right atrium area at 22 cm^2^). Pulmonary artery pressure was mildly elevated at 40 mmHg with pulmonary acceleration time at 98 ms. Mitral flow was in favour of grade II diastolic dysfunction with elevated filling pressure. Pulsed Doppler at mitral valve showed an exclusive E wave of increased velocity (0.73 m/s), a deceleration time of 92 ms, and E/e’ ratio of 12.3, with an E’ velocity at 5.9 cm/s.

To further investigate the syncope aetiology, we performed a cerebral CT scan and supra-aortic trunks echo-Doppler that turned out to be normal. A Holter-ECG found a focal atrial tachycardia with an average heart rate of 103 bpm, frequent premature ventricular complexes (PVC) with bigeminism. Coronary arteries angiography was normal.

**Fig. 3:**
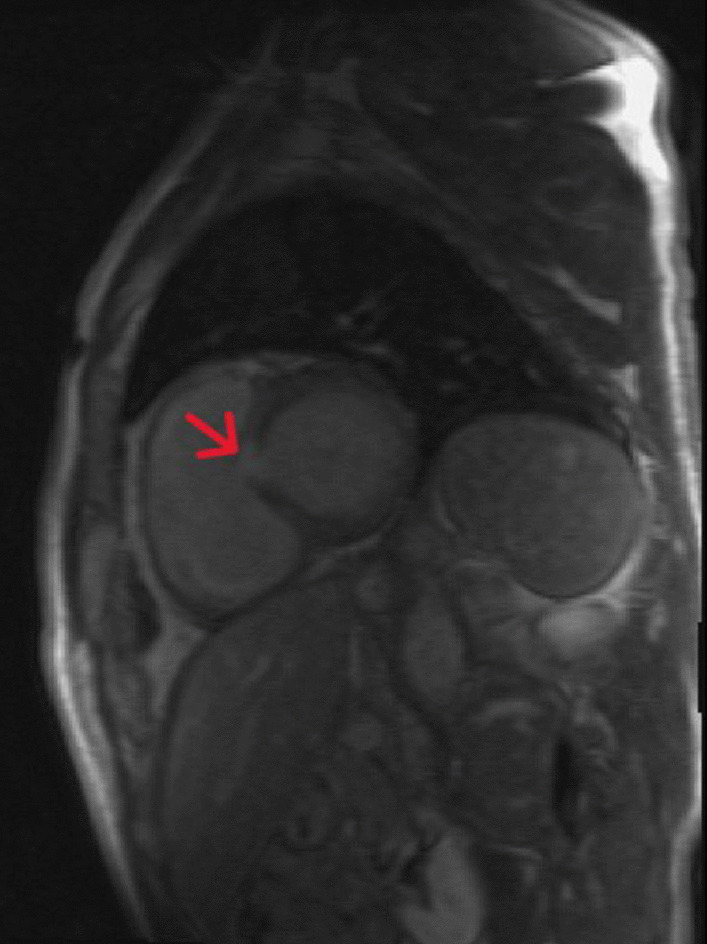
Short axis view showing a Late gadolinium enhancement after 10 min of injection in the basal segment of the infero-septal wall (red arrow)

To rule out a myocardial infiltration, a CMR was performed. It revealed a non-dilated left ventricle (EDV 58 ml/m^2^) with hypertrophy limited to the basal segment of the infero-septal wall with a thickening of 12mm (ventricular mass at 74g/m^2^), a mildly reduced ejection fraction (40%) and global hypokinesis. Late gadolinium enhancement (LGE) showed the presence of a focal interstitial fibrosis in the basal LV infero-septal segment (Fig. 3). Marked bi-atrial enlargement was noted. The right ventricle was non-dilated with normal systolic function.

We hypothesized that the primary mechanism of the syncope was purely arrhythmic. A sudden heart rate acceleration at the initiation of the tachyarrhythmia remains a plausible cause. While a continuous in-hospital heart rate monitoring and a Holter-ECG found a persistent tachycardia with no conduction disturbance. Thus, a cardiac pause at the termination of the tachycardia is of lesser probability.

Given the bi-atrial enlargement and the tachyarrhythmia, an anticoagulation regimen based on apixaban was initiated. The haemorrhagic risk assessment based on HASBLED score (2/9) was moderate, despite the thrombocytopenia which remains above 50.000/mm^3^. Rate control was successfully obtained with beta-blockers. Nonetheless, because of hyperthyroidism, amiodarone could not be initiated. The patient was discharged after an effective diuretic decongestion and normalization of BNP level. He was then referred to a metabolic disease centre to start enzyme replacement therapy (ERT). The patient will benefit from radioiodine therapy for a toxic thyroid nodule revealed by scintigraphy.

## Discussion

Gaucher disease (GD) is the most prevalent lysosomal storage disease that leads to the accumulation of glucocerebroside in reticuloendothelial cell, thus haematological, skeletal and abdominal organs. It is an autosomal recessive disease secondary to a deficit in glucocerebrosidase. Its incidence rate is 1/50000 in the general population but could reach 1/800 among Ashkenazi Jews [2]. Three major clinical sub-types are described depending on the absence (Type I) or presence of neurologic involvement (Type II and III). Type 1 of GD is the most frequent, especially within the Ashkenazi, evolving from adult-onset disorders with major haematological and bony manifestations to childhood disorders, with mainly visceral and bony involvement. Type II is the infant subtype, seen within the first year of life, with acute neurologic involvement and severe prognosis [3]. GD Type III is another form of GD with a subacute neurological involvement associated with visceral findings.

Concerning cardiac involvement, it is rare and mainly reported in type IIIc GD, a subtype described in Spanish and Arab populations, in patients with D409H mutation. It is characterized by extensive calcification of the aorta and leaflets valve [1, 4]. Constrictive calcific pericarditis is a rare cardiac manifestation resulting from intrapericardial haemorrhage related to thrombocytopenia seen in GD [5]. Coexistence of Gaucher disease with restrictive cardiomyopathy is rare [6].

It is important to evaluate pulmonary artery hypertension, which is rare but well-described in type I GD [7] with an incidence rate of up to 20%, based on Doppler echocardiographic assessment [8]. According to the European Association of Cardiovascular Imaging criteria to assess the probability of PAH, our patient presented mild pulmonary hypertension with an estimated systolic PAP (sPAP) of 40mmH. Similar results were reported in a large Romanian cohort with mean sPAP of 41mmHg, found in 13% of patients [9]. Risk factors of symptomatic PAH in GD are female sex, splenectomy, non-N409S GBA mutation, angiotensin-converting enzyme gene polymorphism and family history. None of those is present in our case.

The diastolic function may be compromised due to infiltrative processes leading to left ventricular hypertrophy and patchy interstitial fibrosis [10]. In our study, Doppler assessment found a grade II diastolic dysfunction with elevated filling pressure; however, E/e’ ratio was normal. Neither E/A ratio nor A mitral wave could be studied due to Atrial arrhythmia.

Only two studies were interested in evaluating diastolic function in Type 1 GD. Koželj and al. reported a tendency towards dysfunction with no statistically significant difference between patients and the control group [11]. However, Lo Iudice investigated LV geometry and function in 18 GD patients compared to hypertensive and normal subjects. A prolonged deceleration time of E wave velocity, greater atrial filling fraction and normal E/e’ ratio were observed. There is a pattern of impaired relaxation with normal filling pressure [12]. Worthy of note, all GD were under enzyme replacement therapy, which partly explains the absence of LV hypertrophy.

We hypothesize that our patient had an impaired diastolic function, recently decompensated by the onset of tachyarrhythmia.

Besides LV hypertrophy and bi-atrial enlargement, our patient presented a global hypokinesis with a regional wall motion abnormalities in basal and middle infero-septal and inferior segments. Alizad described similar findings in adult GD patients, with increased LV mass and a septal muscular prominence [13].

CMR was performed in order to study myocardial infiltration. Recently, Solanich assessed the GD cardiac involvement of a 62-year-old type I GD patient with no cardiac symptoms using CMR. LGE showed multiple foci consistent with interstitial infiltrative fibrosis in the basal and middle inferior and inferolateral segments, in addition to bi-atrial enlargement [14]. Similar findings are seen in our case, even if LGE was only located in the basal infero-septal segment. Without an objective histological examination, the focal interstitial fibrosis may correspond to a local reaction in response to sphingolipid infiltration.

In a larger cohort of patients with type I GD, no interstitial fibrosis was reported neither in patients on ERT nor at diagnosis. Atrial enlargement is present even in the absence of structural heart disease. An accurate assessment of diastolic function and interstitial fibrosis is warranted by using a multi-modal approach with the aid of CMR imaging and echocardiography evaluation [8].

Enzyme replacement therapy is the cornerstone for treating GD with a positive outcome regarding anaemia, thrombocytopenia, and organomegaly [3]. Concerning cardiac involvement, Spada reported an improvement of LV systolic and diastolic function and normalization of T-wave within months of a high dose of ERT, and complete normalization of echocardiogram after 3 years [15].

Considering the absence of myocardial infiltration in GD on ERT in the cohort of Roghi et al. [8], ERT may have a significant role in preventing cardiac manifestations. Nonetheless, its effect in treating valve calcification is debatable. As reported by previous cases, it did not seem to stop calcification progression [1, 16].

## Conclusions

Even though cardiac involvement of GD has been previously reported especially in particular genotypes, further studies should focus on cardiac abnormalities to establish an imaging hallmark of GD. We recommend using a shared dataset of the International Collaborative Gaucher Group Registry, which may help clarify their incidence and provide additional therapeutic goals.

Non-invasive cardiac imaging, by both CMR and echocardiography, can therefore provide essential information regarding diagnosis, prognosis, and management of cardiac manifestations of GD.

## Data Availability

All data and materials related to this report are accessible at any time upon request.

## Abbreviations

GD: Gaucher disease
CMR: Cardiac magnetic resonance
LVH: Left ventricular hypertrophy
PVC: Premature ventricular complex
LGE: Late gadolinium enhancement
ERT: Enzyme replacement therapy
PAH: Pulmonary arterial hypertension
sPAP: Systolic pulmonary arterial pressure

## References

[1] Kör Y, Keskin M, Başplnar O (2017). Severe cardiac involvement in Gaucher type IIIC: a case report and review of the literature. Cardiol Young.

[2] Nguyen Y, Stirnemann J, Belmatoug N (2020). Maladie de Gaucher. Rev Prat.

[3] Grabowski GA (2008). Phenotype, diagnosis, and treatment of Gaucher’s disease. Lancet.

[4] Brautbar A, Abrahamov A, Hadas-Halpern I, Elstein D, Zimran A (2008). Gaucher disease in Arab patients at an Israeli referral clinic. Isr Med Assoc J.

[5] Chabás A, Cormand B, Grinberg D, Burguera JM, Balcells S, Merino JL (1995). Unusual expression of Gaucher’s disease: cardiovascular calcifications in three sibs homozygous for the D409H mutation. J Med Genet.

[6] Kundu S, Dasgupta MK, Majumder B, Pradhan S (2021). Restrictive cardiomyopathy: a rare presentation of Gaucher disease. Ann Afr Med.

[7] Mistry PK, Sirrs S, Chan A, Pritzker MR, Duffy TP, Grace ME (2002). Pulmonary hypertension in type 1 Gaucher’s disease: genetic and epigenetic determinants of phenotype and response to therapy. Mol Genet Metab.

[8] Roghi A, Poggiali E, Cassinerio E, Pedrotti P, Giuditta M, Milazzo A (2017). The role of cardiac magnetic resonance in assessing the cardiac involvement in Gaucher type 1 patients: morphological and functional evaluations. J Cardiovasc Med.

[9] Lazea C, Bucerzan S, Al-Khzouz C, Zimmermann A, Vesa C, Nas I (2021). Cardiac Manifestations in a group of romanian patients with Gaucher disease type 1 (a monocentric study). Diagnostics.

[10] Smith RRL, Hutchins GM, Sack GH, Ridolfi RL (1978). Unusual cardiac, renal and pulmonary involvement in Gaucher’s disease. Am J Med.

[11] Koželj M, Zver S, Zadnik V (2010) Echocardiographic assessment of left ventricular function in type 1 Gaucher’s disease. Adv Hematol 2010 10.1155/2010/820843 PMC2913527 20721274

[12] Lo Iudice F, Barbato A, Muscariello R, Di Nardo C, De Stefano F, Sibilio M (2015). Left ventricular diastolic dysfunction in type i gaucher disease: an echo doppler study. Echocardiography.

[13] Alizad A, Seward JB (2000). Echocardiographic features of genetic diseases: part 5. Tumors J Am Soc Echocardiogr.

[14] Solanich X, Claver E, Carreras F, Giraldo P, Vidaller A, Aguilar R (2012). Myocardial infiltration in Gaucher’s disease detected by cardiac MRI. Int J Cardiol.

[15] Spada M, Chiappa E, Ponzone A (1998). Cardiac response to enzyme-replacement therapy in Gaucher’s disease. N Engl J Med.

[16] Saleh Y, Almaghraby A, Hammad B, Mokhtar A, Abdel-hay MA (2016). Gaucher disease causing sudden cardiac death. Egypt Hear J.

